# Pilot-Scale Aluminothermic Production of Silicon Alloy and Alumina-Rich Slag

**DOI:** 10.1021/acssuschemeng.4c05326

**Published:** 2024-09-10

**Authors:** Mengyi Zhu, Harald Philipson, Kjell Blandhol, Veronika Djupvik, Krister Engvoll, Jafar Safarian, Kai Tang, Mårten Görnerup, Elisa Pastor-Vallés, Maria Wallin, Gabriella Tranell

**Affiliations:** † Department of Materials Science and Engineering, Norwegian University of Science and Technology (NTNU), 7491 Trondheim, Norway; ‡ Elkem Technology, 4621 Kristiansand, Norway; § SINTEF Industry, N-7465 Trondheim, Norway; ⊥ Department of Energy and Process Engineering, Norwegian University of Science and Technology (NTNU), 7491 Trondheim, Norway

**Keywords:** Silicon, Alumina, Aluminothermic reduction, Secondary materials, Slag

## Abstract

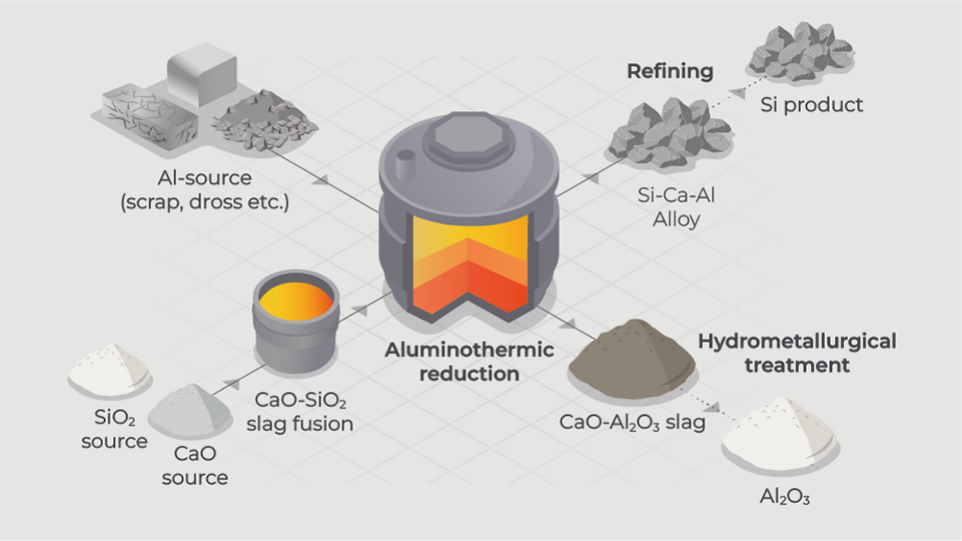

As global demand for silicon (Si) and alumina continues to surge, the significance of developing more sustainable production methods has intensified. The SisAl process represents a path-breaking approach to producing Si and alumina by utilizing side streams from the silicon and aluminum industries. In the present work, a comprehensive series of pilot-scale trials was conducted to assess the scalability and validity of the SisAl process. The influences of reductant raw materials, charging methods, input material ratios, and other processing parameters were also investigated. On coupling between thermodynamic calculations and experimental results, it was demonstrated that the SisAl process is stable and controllable at a pilot scale. The typical composition of the produced Si alloy ranges from 70 to 75 wt % Si, 15–18 wt % Ca, and 8–10 wt % Al. The produced CaO-Al_2_O_3_-based slag has a composition of 48–57 wt % Al_2_O_3_, 40–45 wt % CaO, and 3–13 wt % SiO_2_. Furthermore, various reductants, including Al blocks, dross, and scraps, were evaluated in the pilot trials, yielding comparable outcomes, with alloy composition differences only within approximately 3 wt % under the same parameter conditions, demonstrating the versatility of raw materials selection in the SisAl process. Moreover, it was found that acidic slag exhibited better features for future upscaling than the neutral slags, with a 5 wt % higher Si content in the alloy and a 4 wt % increase in Al_2_O_3_ content in the slag phase. Additionally, the chemical composition of the products was shown to be controllable by adjusting the stoichiometry of the input materials (Al/SiO_2_ ratio). As the charged stoichiometry increases from 1 to 1.2, the Si content in the alloy significantly decreases from 73 to 63 wt %, while the Al content correspondingly increases from 9 to 18 wt %. Further investigation into processing parameters, such as charging method, slag prefusion, and stirring methods, has also revealed valuable insights toward the continued upscaling of the SisAl process to a potentially innovative, sustainable, low-carbon industrial process.

## Abstract 2

A path-breaking approach has been developed for sustainable silicon and alumina production from aluminothermic reduction through industrial symbiosis between Si and Al industries.

## Introduction

Silicon (Si) and aluminum (Al), the second and third most abundant elements in the Earth’s crust, are critical to the technological and industrial advancement of the global community. They are in high demand for a wide variety of essential applications ranging from electronics and solar panels to lightweight automobiles and aircrafts. Moreover, in the coming decades, the clean energy transition to mitigate climate change will also bring accelerated demands on these primary raw materials. It is expected that the solar photovoltaic (PV) industry will continue to grow rapidly with an average annual installation growth of 25% over the period 2022–2030 and a 12 times increase in solar silicon production capacity is required to achieve net-zero emissions by 2050.^1,2^ In parallel, the rapid acceleration of the global electric vehicle market has become one of the main driving forces for boosting the Al production;^3^ accordingly, the global Al demand is forecast to grow to 119.5 Mt in 2030.^4^

However, the current production of the two materials is faced by sustainability concerns due to their associated environmental impacts. Metallurgical grade silicon (MG-Si, >98% Si) is exclusively produced by the traditional submerged arc furnace (SAF) process, which involves the carbothermic reduction of quartz at high temperature.^5^ This process inherently leads to greenhouse gas emissions and is associated with high energy consumption.^6^ Meanwhile, alumina, as the direct source of Al, is dominantly produced from bauxite through the Bayer process, which generates large amounts of red mud, a highly alkaline waste resulting in both environmental and disposal challenges. Presently, red mud is not effectively utilized on a large scale and the overall red mud accumulation has surpassed 4 billion tons worldwide.^7,8^ Besides red mud, white dross,^9^ another byproduct of the aluminum industry, forms due to the surface oxidation of molten aluminum and also adds to the waste management issues and requires proper treatment for recycling and utilization.

**Figure 1:**
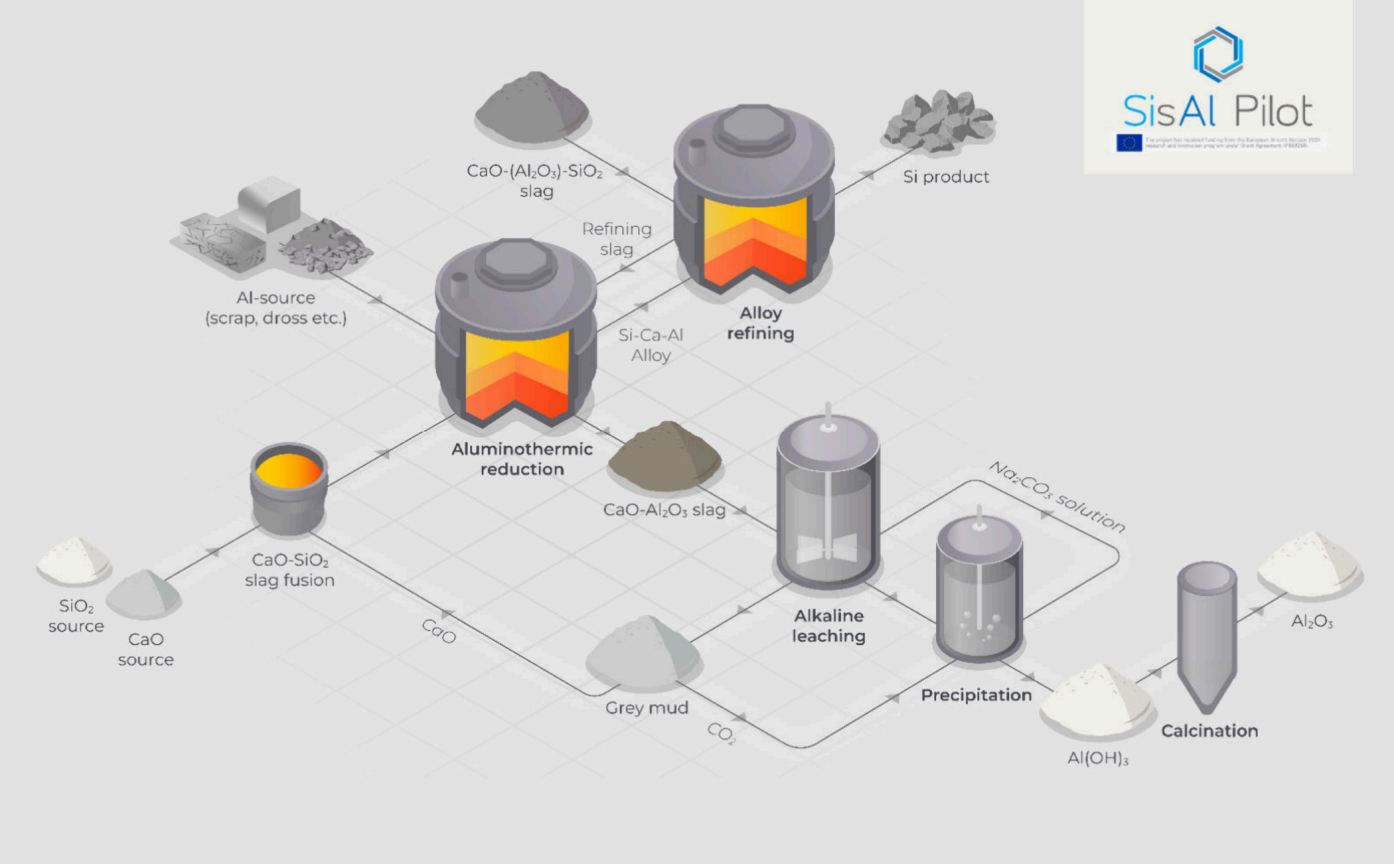
Schematic representation of the SisAl process illustrating the aluminothermic reduction using a secondary aluminum source and CaO-SiO_2_ slag for the production of silicon and alumina. The alloy refining techniques presented may vary based on the desired silicon grade, such as MG-Si, RMG-Si, SoG-Si, and hypereutectic Al–Si alloy. The produced CaO-Al_2_O_3_-based slag undergoes hydrometallurgical treatments to produce various grades of alumina such as MGA and HPA.

In response to the aforementioned pressing issues, the SisAl process (Silicon production using secondary Aluminum, www.sisal-pilot.eu)^10,11^ was developed as an innovative and environmentally friendly method for the sustainable production of silicon and alumina. As depicted in Figure 1, instead of using the conventional carbon reductants, the SisAl process utilizes secondary raw materials such as Al scrap and Al dross to produce various grades of Si, Si-Al alloys, and alumina via aluminothermic reduction of SiO_2_ in a CaO-SiO_2_ slag following the overall reaction
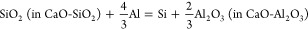
1

The aluminothermic reduction products comprise a Si-Ca-Al alloy as the metal phase and a CaO-Al_2_O_3_-based slag as the slag phase. Through a subsequent slag refining procedure, the Si alloy can be further upgraded to different commercial grades of silicon products,^12^ such as MG-Si (target purity +99%), refined MG-Si (RMG-Si, target purity +99.9%), SoG-Si (target purity +99.9999%), and hyper-eutectic Al-Si alloys (target purity <0.3% Fe). The slag further undergoes hydrometallurgical treatments^13,14^ to produce metallurgical-grade alumina (MGA, target purity +99%) or high-purity alumina (HPA, target purity 99.99%).

In contrast to the Bayer process, which generates red mud as industrial waste, the SisAl process generates a CaCO_3_-based grey mud that can be recycled back into the process to form a closed materials flow. The calcined grey mud residue is recycled as the CaO source for the prefusion of CaO-SiO_2_ slag, while the resulting CO_2_ gas can be used for the precipitation of Al(OH)_3_.^15^ Additionally, compared to the current SAF process for Si production, the reduction of SiO_2_ in the SisAl process occurs with molten CaO-SiO_2_ slag, lowering the reaction temperature by several hundred degrees. The molten slag also offers enhanced kinetic conditions, and the exothermic nature of the thermite reaction provides energy efficiency benefits due to the substantial heat released. Furthermore, since the SisAl process features a liquid–liquid reaction, it is no longer necessary to impose restrictions over the raw materials’ mechanical properties, such as quartz strength and particle size. This characteristic substantially increases the flexibility of the raw materials selection; for instance, the process allows for the use of quartz fines. It can also facilitate the recycling of Si sculls and slags. Thus, by utilization of waste from the silicon and aluminum industries, the SisAl process introduces an innovative industrial symbiosis model that promotes a low-carbon circular economy.

In our previous work, the feasibility,^16^ variability of raw materials,^17^ and technoeconomic considerations^10^ have been demonstrated. Recently,^17^ the effects of using different sizes and types of Al dross as reductants in the SisAl process were also investigated. In the 100 g- and kg-scale experiments, the produced Si alloys contains 66–83 wt % Si under various reaction parameters. Additionally, recent findings showed that the fast reduction kinetics were primarily driven by enhanced mass transfer and interfacial area due to the interfacial turbulence at higher temperatures (1600–1650 °C).^18^ Moreover, in a recent kinetics study for an Al-SiO_2_ stoichiometry of 1, dropping solid Al into non-agitated liquid CaO-SiO_2_ slag at 1650 °C achieved conversions relative to thermodynamic equilibrium of 72% for Ca and 83% for Si and Al within only 15 s.^19^

The focus of the present work is to test different materials and conditions on a larger scale to provide a more robust response and indication process variability, aiming to bridge the gap between laboratory research and industrial application through a series of pilot-scale trials. The pilot scale trials were designed and conducted to provide valuable insights into the scalability and validity of the SisAl process. In the pilot trials, the effects of various operational parameters such as raw materials type, charging methods, and input raw materials ratios were investigated. An in-depth thermodynamic analysis of the pilot results was performed in an effort to contribute to the further development of this innovative process and bring us one step closer to achieving a sustainable industrial process.

## Materials and Methods

A total of 18 pilot-scale aluminothermic reduction trials were conducted by Elkem Technology R&D Center in Kristiansand, Norway. The effort was aimed at demonstrating the scalability of the SisAl process and represented a significant advancement from the previously successful lab-scale tests.^10,17^

The trials were divided into a series of subcampaigns, with each subcampaign encompassing multiple individual trials. Three different reductant types were evaluated: Al blocks, compact Al scrap, and Al dross (Table 1). Moreover, the impacts of various factors in the process, including material charging methods, stirring methods, slag basicity, and the reactant charging ratio based on reaction stoichiometry (4 Al/3 SiO_2_) were also evaluated. For each set of process conditions, two parallel tests were performed.

**Table 1:** Parameters of the Pilot SisAl Process Pilot Trials

	reductant		concentrate							
test ID	type of Al	amount (kg)	prefused CaO-SiO_2_ slag (kg)	quartz (kg)	lime (kg)	total quantity (kg)	basicity	reactant stoichiometry (4/3 Al/SiO_2_)	charging type	stirring method
T1	block	109	175	87.5	87.5	459	1	1	sprinkle	gas
T2	block	109	175	87.5	87.5	459	1	1	sprinkle	gas
T3	block	109	175	87.5	87.5	459	1	1	sprinkle	rotor+gas
T4	block	109	175	87.5	87.5	459	1	1	sprinkle	rotor+gas
T5	block	109	175	87.5	87.5	459	1	1	co-charging	gas
T6	block	109	175	87.5	87.5	459	1	1	co-charging	gas
										
T9	dross	150	350	-	-	500	1	1	sprinkle	gas
T10	dross	150	350	-	-	500	1	1	sprinkle	gas
T11	dross	150	350	-	-	500	1	1	co-charging	gas
T12	dross	150	350	-	-	500	1	1	co-charging	gas
										
T7	scrap	144	310	39	-	493	0.8	1.2	co-charging	gas
T8	scrap	144	310	39	-	493	0.8	1.2	co-charging	gas
T17	scrap	111	50	150	150	461	1	1	sprinkle	gas
T18	scrap	111	50	150	150	461	1	1	co-charging	gas
T19	scrap	144	250	68.7	31.4	494	1	1.2	sprinkle	gas
T20	scrap	131	250	51	45.4	477	1	1.2	sprinkle	gas
T21	scrap	131	250	51	45.4	477	1	1.2	sprinkle	gas
T22	scrap	131	250	51	45.4	477	1	1.2	sprinkle	gas

Most of the trials utilized an initial slag composition with a 1:1 weight ratio of SiO_2_ to CaO, which exhibited favorable melting properties and low viscosity. In all trials, the liquid–liquid aluminothermic reaction occurred at a temperature ranging between 1550 and 1650 °C, resulting in the formation of a Si alloy and an alumina-rich CaO-Al_2_O_3_-SiO_2_ slag, which were subsequently separated through tapping. Both the silicon alloy and slag were characterized in terms of composition and mass. Key performance indicators were established and associated with relevant metallurgical parameters, serving to evaluate the overall success and efficiency of the process.

### Raw Materials

**Figure 2:**
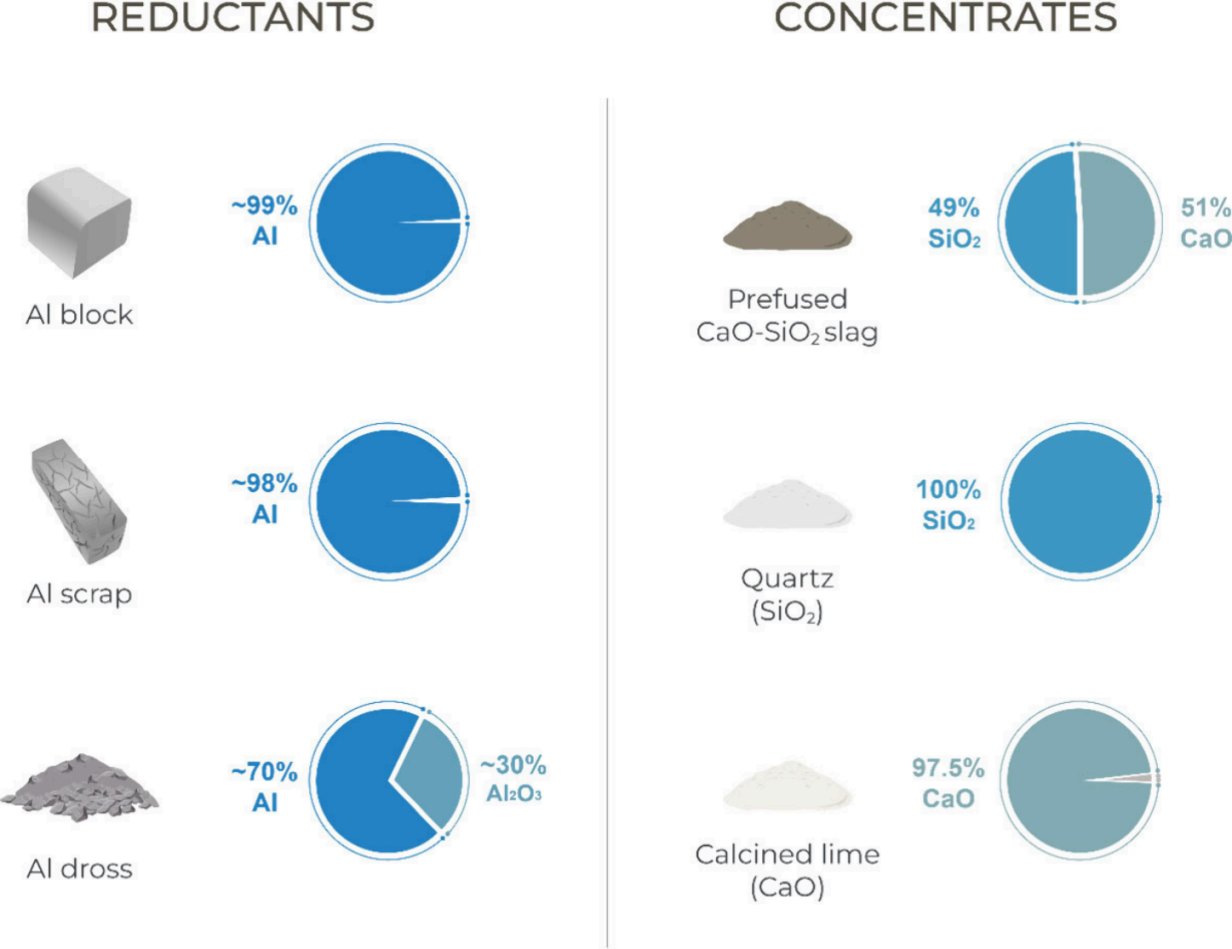
Compositions of raw materials utilized in the pilot trials.

The types of input materials utilized and their respective compositions are listed in Figure 2. The Al blocks (98.7% purity, 0.53% Si, 0.45% Mg, and 0.20% Fe) are considered nearly pure metal. The Al scrap, composed primarily of compact scrap turnings, contains about 98.3% metallic Al, with the main impurity being 0.78% Mg, 0.59% Si, and 0.20% Fe. The Al dross, provided by an industrial partner, was estimated from our previous work to contain approximately 70% metallic Al content.^17^ The CaO-SiO_2_ slag for the aluminothermic reduction was prefused by incorporating 51 wt % CaO and 49 wt % high-purity quartz. The fused slag was subsequently cast and crushed into lumps less than 50 mm in size. During the pilot trials, specific quantities of quartz and calcined CaO were also charged. In addition, prior to being charged into the furnace, all oxides were heated at 80 °C for a period of 12 h.

### Equipment

**Figure 3:**
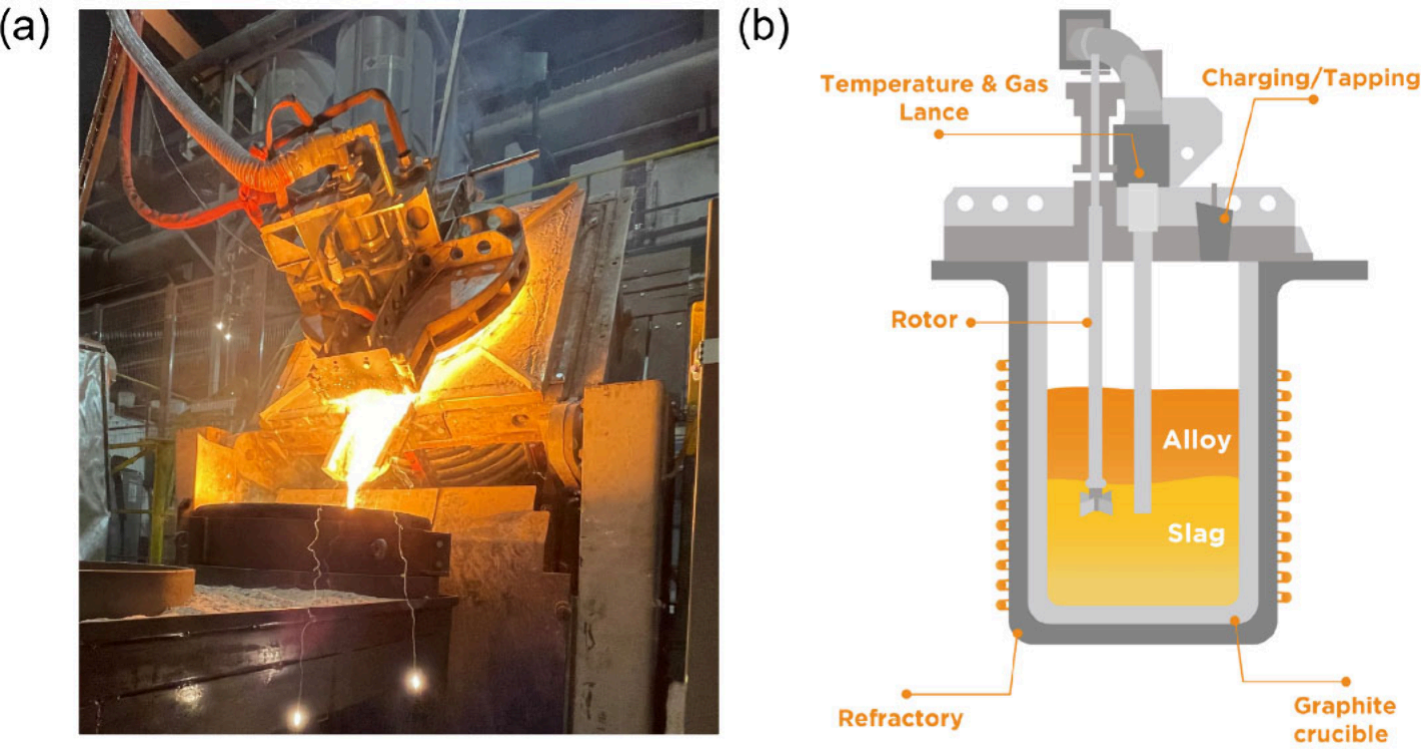
(a) Real image and (b) schematic illustration of the furnace structure showing dimensions and component positions.

The pilot-scale trials were conducted by utilizing a 600 kW induction furnace, outfitted with a water-cooling system. Figure 3 provides a visual representation of the furnace, detailing the key components and dimensions. The depicted height of the slag and metal melt is approximate, while the depth of the rotor and lance is scaled relative to the slag level and crucible height.

Within the furnace, a graphite crucible was housed with a volumetric capacity of 234 L (inner diameter 550 mm, depth 985 mm, height 1035 mm). The furnace featured a removable lid that housed a small hatch for manual charging. This lid was manipulated by a custom-built robot to ensure precision and safety prior to the tapping process. The tapping process itself was facilitated through a hydraulic furnace tilting system.

The operational power was modulated between 120 and 180 kW to maintain the melt temperature within the range 1550–1650 °C, while the temperature was measured by a Type-C thermocouple positioned near the center of the slag melt both vertically and horizontally. The melt was primarily agitated by purging nitrogen gas through a top lance in addition to the induction-induced melt movement. In two specific trials, in addition to gas purging, the melt was mechanically stirred by using a rotor. Both the rotor and lance were also made of graphite. Gas injection was started after the last addition of the Al and applied for the entire duration of the reaction and holding stage with a flow rate between 20 and 25 L/min. The thermocouple and purging inlet were parallel and approximately 27 cm from the bottom. The gas lance and crucible were replaced regularly due to wear at the part of the lance closest to the melt surface.

### Operation

**Figure 4:**
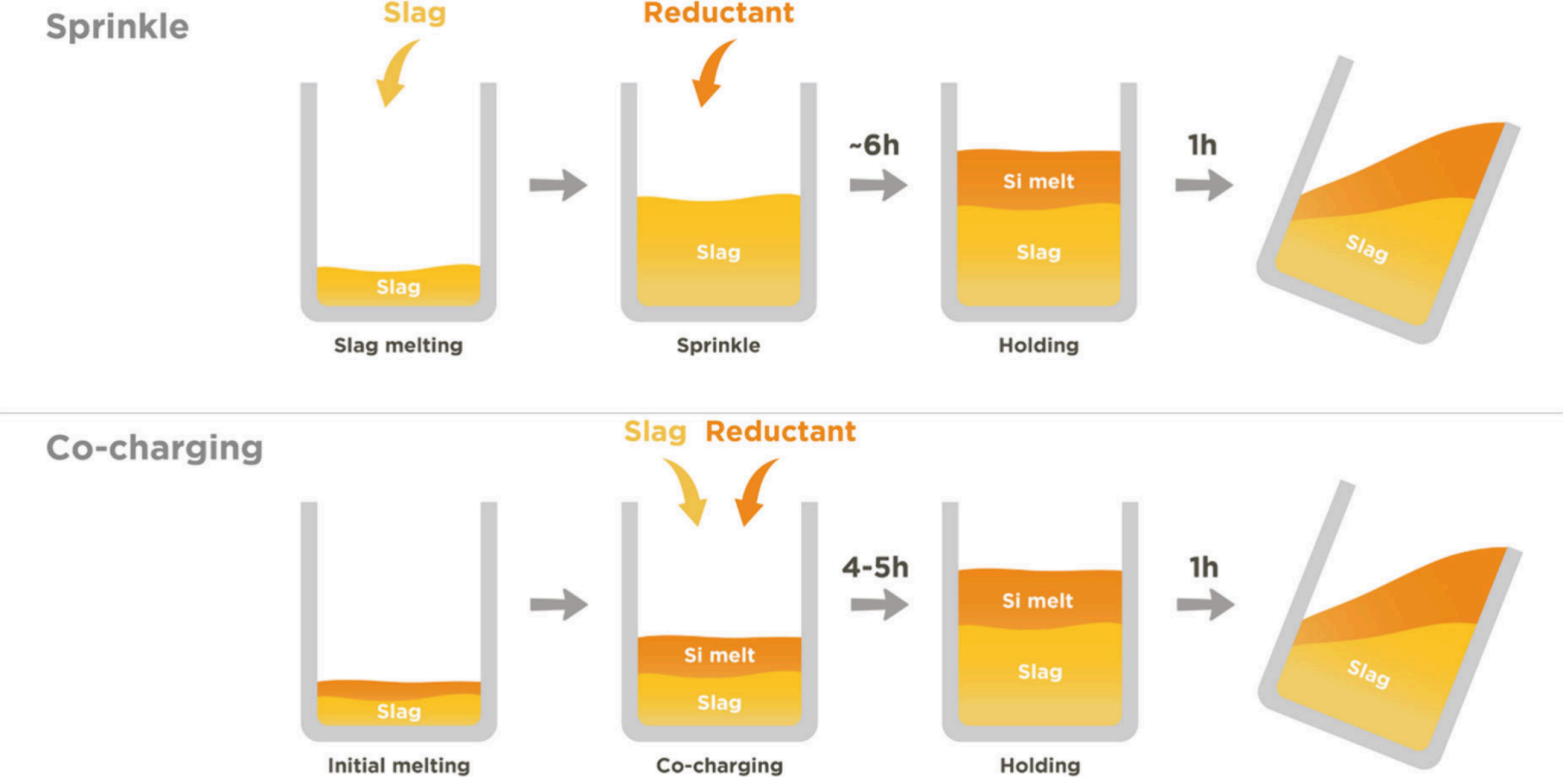
Sketch of the investigated charging methods: (1) sprinkle and (2) co-charging. The presented time lengths are only applicable for this pilot test and are not indicative of final production durations.

As illustrated in Figure 4, two distinct charging methods were tested in the pilot trials, respectively: sprinkle charging, which involves slag melting prior to the addition of an Al source, and co-charging, which includes the simultaneous charging of slag and the Al source.

In both methods, the process was initiated with the formation of a molten heel of CaO-SiO_2_ slag at the bottom of the furnace. This was achieved by applying power up to 150 kW for a duration of approximately 2 h until a temperature of 1600 °C was attained. For the sprinkle method, the subsequent steps included (1) gradual addition of the total 350 kg of slag in portions every 10–20 min, ensuring the complete melting of each previous addition, (2) once all the slag had been introduced and melted, the reductant (ranging between 109 and 150 kg) was added in 5–8 kg increments over a span of approximately 90 ± 20 min, (3) the furnace was then held at the target temperature for 1 h to facilitate the reaction, and finally, (4) tapping the Si alloy followed by the slag into 4 to 5 graphite molds, for approximately 1 h of time.

In contrast, the co-charging method involves varying amounts of addition of reductant and slag together each time, contingent upon the type of reductant utilized. The specific combinations used were 3–7 kg of Al blocks with 10 kg of slag, 5–8 kg of scrap with 10 kg of slag, or 7.5 kg of dross with 15 kg of slag. Each new charge was added once the previous one had completely melted, and the total charging process typically spanned 4–5 h. Finally, the furnace was, as in the sprinkle method, held at the target temperature for 1 h prior to the tapping process.

The tapping process is seen in Figure 3a. The gas was reduced to 2–3 L/min during tapping in most trials and 0 L/min for the last four trials. Sparks appeared only for tapping of the alloy, which allowed the operator to distinguish between the metal and slag phases. It is noteworthy that some tapped slags entrained alloy droplets varying in size from micrometers to centimeters. To ensure a more reliable composition measurement, the Fedorov analysis^20^ was utilized for representative slag samples.

### Sampling and Chemical analysis

To evaluate the pilot trials, liquid slag samples were obtained by immersing a graphite tube 20–30 cm into the melt. The molten slag solidified on the tube upon extraction. As the tube had to first penetrate through the alloy layer, a thin layer of the alloy also inevitably adhered to the tube. About 150 g of liquid samples (up to 10 samples) was taken during the operation. This collected sample was then carefully fragmented and milled down to a particle size suitable for X-ray fluorescence (XRF) analysis, which employed the Wavelength Dispersive XRF (WD-XRF) technique on glass-fused beads (utilizing LiBO_2_ and oxidizers) via the FESIFLUX method.

Furthermore, solid samples were taken from the cast alloy and slag. For consistency, samples close to the edge of the cast were selected, each weighing approximately 250 g. These samples were subsequently milled in preparation for XRF analysis.

## Results

### Composition of Produced Alloy and Slag

**Figure 5:**
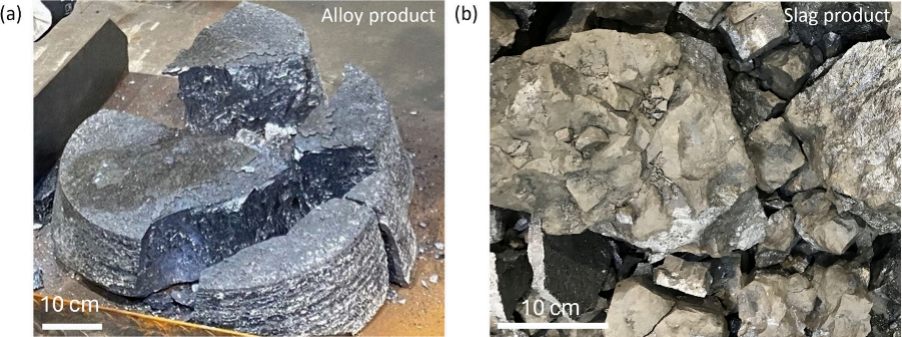
Example images of pilot products: (a) SisAl silicon alloy and (b) alumina-rich SisAl slag.

In each trial, the molten alloy was first tapped, followed by tapping of molten slag. The typical alloy and slag products are displayed in Figure 5.

**Figure 6:**
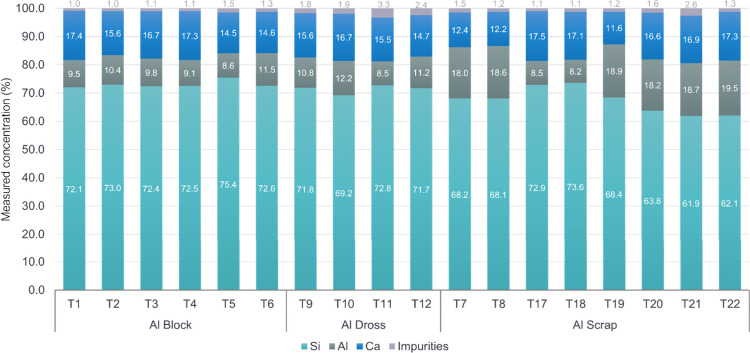
Composition of the produced Si alloys after aluminothermic reduction. Values are given in weight percent (wt %).

**Figure 7:**
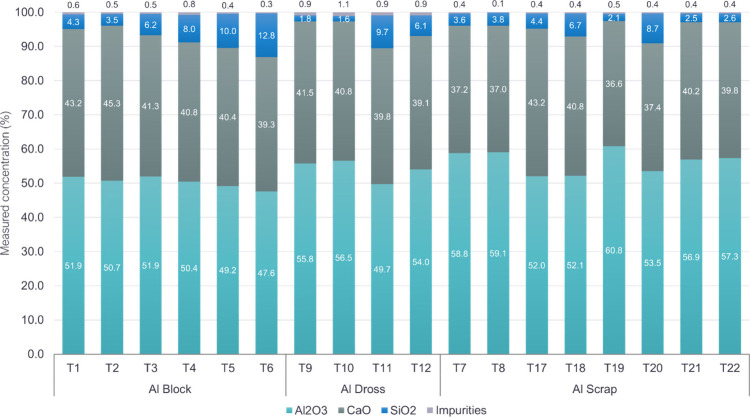
Composition of the produced Al_2_O_3_-rich slag after aluminothermic reduction. Values are given in weight percent (wt %).

The chemical composition of produced alloys and slags were examined through XRF analysis, and the results are presented in Figures 6 and 7. The alloy compositions exhibited high similarity with Si consistently being the dominant element and the concentrations of Ca and Al varying slightly. In addition, it also indicates that Si has the highest variability in its composition, which ranges from the lowest (61.9 wt %) to the highest (75.4 wt %) depending on different conditions. The variability of Ca and Al was within ranges, respectively, from 11.6 to 17.5 wt % for Ca, and from 8.2 to 19.5 wt % for Al. The impurity content in the produced alloy ranges from approximately 1 wt % up to 3.3 wt %, largely depending on the type of reductant used. In trials utilizing dross, impurity levels range from about 1.8 to 3.3 wt %, with primary impurities including approximately 0.8 wt % Fe, 0.5 wt % Mg, and 0.3 wt % Mn. When using Al blocks, the contents of Fe and Mg decrease to around 0.3 and 0.2 wt %, respectively.

The slag composition falls within the range feasible for subsequent hydrometallurgical treatment for Al extraction.^21^ The content of Al_2_O_3_ was found to range between 47.6 and 53.8 wt %, and the CaO content exhibited a range from 36.6 to 45.3 wt %. The SiO_2_ content showed a broader variation, with its content fluctuating between 1.6 and 12.8 wt %, most likely due to differences in sample areas and the presence of Si alloy inclusions in the slag. The primary impurities in the slag were detected as MgO and Fe_2_O_3_, each approximately around 0.3 wt %.

### Microstructure of Produced Alloy and Slag

**Figure 8:**
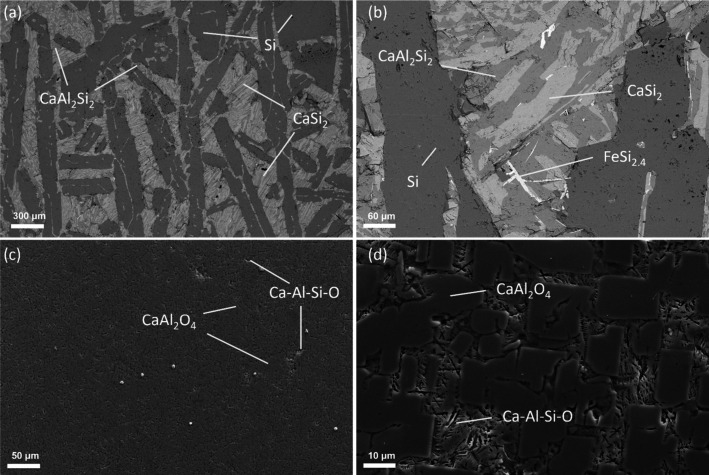
Microstructure and phases in the aluminothermic reduction producing Si alloy (a, b) and aluminate slag (c, d) from Trial 5.

As a representative sample, the microstructure of the alloy and slag produced in T5 are presented in Figure 8. The produced Si–Ca–Al alloy consists of a Si matrix formed by the primary Si and a eutectic phase region with the appearance of two silicides, CaSi_2_ and CaAl_2_Si_2_. The primary Si is plate-like with a thickness ranging from 100 to 200 μm. The CaSi_2_ and CaAl_2_Si_2_ formed after the primary Si formation are seen to form a typical eutectic pattern. Additionally, it is also seen that a needle-like Fe-bearing phase is also embedded inside the eutectic region. Through a subsequent slag refining process, most impurities can be rapidly eliminated, allowing the Si-Ca-Al alloy to be further refined to achieve 99 wt % Si.^22^ Alternatively, the Si-rich alloy can also be further upgraded to MG-Si through acid leaching. This is due to the high leachability of both the CaSi_2_ and CaAl_2_Si_2_ phases, which allows for the efficient removal of the eutectic phase and the impurities embedded inside.^23^

Since the cooling rate was not controlled, a minor glassy phase formed at the edge regions after casting, but the majority of the cooled slag consisted of precipitated crystalline oxides with a typical microstructure that contains the krotite phase (CaAl_2_O_4_) and a secondary Ca-Al-Si-O-containing phase with a higher concentration of Si. Under the appropriate conditions, mayenite (Ca_12_Al_14_O_33_) and tricalcium aluminate (Ca_3_Al_2_O_6_) can also be formed. All of the aforementioned calcium aluminates can be readily dissolved in sodium carbonate or hydrochloric solution for the extraction of Al and subsequent alumina production of MGA or HPA.^21,24^

### Operation Performance

In Table 2, the average output quantity, composition, and operation indices of each subcampaign are calculated and listed. In this study, the Al yield is defined as the percentage of reacted Al during the aluminothermic smelting. It represents the raw material utilization efficiency by expressing the consumed Al to total Al mass. In this work, for simplicity and to minimize the effect of potential slag composition measurement error caused by the entrapped alloy droplets, the Al yield is expressed as the complement of the percentage representing the residual Al in the metal phase with respect to the initial charged mass of metallic Al. For further insights into the materials flow from both technical and economic perspectives as well as Life Cycle Assessment (LCA), the reader is referred to refs (10 and 25).
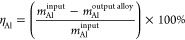
2where *m*_Al_^input^ and *m*_Al_^output alloy^ represent the mass of initial input metallic Al and the mass of Al in the output alloy, respectively.

**Table 2:** Results with Comparison of Multiple Factors on the Reduction Process^a^

	reductant type			charging method		stoichiometry		slag basicity		stirring method		prefused slag/flux ratio	
factors	Al block (T1, T2)	Al dross (T9, T10)	Al scrap (T17, T18)	sprinkle (T1–4, T9, T10, T17, T19–T22)	co-charging (T5–T8, T11, T12, T18)	low Al/SiO_2_ (T17, T18)	high Al/SiO_2_ (T20, T22)	acidic (T7, T8)	neutral (T20, T22)	gas (T1, T2)	gas+rotor (T3, T4)	high prefused slag (T1, 2, T5, 6)	high flux (T17, 18)
output metal (kg)	102 ± 0	99 ± 5	92 ± 3	100 ± 7	103 ± 13	92 ± 3	108 ± 11	115 ± 15	108 ± 11	102 ± 0	95 ± 3	104 ± 2	92 ± 3
Si wt %	72.5 ± 0.4	70.5 ± 1.3	73.3 ± 0.3	69.1 ± 4.3	71.8 ± 2.5	73.3 ± 0.3	62.9 ± 0.9	68.1 ± 0	62.9 ± 0.9	72.5 ± 0.4	72.4 ± 0.1	73.3 ± 1.3	73.3 ± 0.3
Al wt %	10 ± 0.4	11.5 ± 0.7	8.3 ± 0.1	13.2 ± 4.3	12.1 ± 4.1	8.3 ± 0.1	18.8 ± 0.7	18.3 ± 0.3	18.8 ± 0.7	10 ± 0.4	9.5 ± 0.4	10 ± 1.1	8.3 ± 0.1
Ca wt %	16.5 ± 0.9	16.1 ± 0.6	17.3 ± 0.2	16.3 ± 1.6	14.4 ± 1.6	17.3 ± 0.2	16.9 ± 0.4	12.3 ± 0.1	16.9 ± 0.4	16.5 ± 0.9	17 ± 0.3	15.5 ± 1.2	17.3 ± 0.2
Si mass (kg)	74 ± 0.4	69.5 ± 4.5	67.4 ± 1.9	69.5 ± 4.2	73.9 ± 8.7	67.4 ± 1.9	67.5 ± 5.7	78.3 ± 10.2	67.5 ± 5.7	74 ± 0.4	68.8 ± 2.1	76.2 ± 3.1	67.4 ± 1.9
Al mass (kg)	10.2 ± 0.4	11.3 ± 0.2	7.7 ± 0.4	12.8 ± 4.8	12.7 ± 5.7	7.7 ± 0.4	20.3 ± 2.7	21.1 ± 3.1	20.3 ± 2.7	10.2 ± 0.4	9 ± 0.6	10.4 ± 1	7.7 ± 0.4
Ca mass (kg)	16.8 ± 0.9	15.9 ± 0.2	15.9 ± 0.7	16.2 ± 2.1	14.7 ± 1.5	15.9 ± 0.7	18.2 ± 2.2	14.1 ± 1.8	18.2 ± 2.2	16.8 ± 0.9	16.1 ± 0.2	16.1 ± 1	15.9 ± 0.7
output slag (kg)	351 ± 22	385 ± 18	356 ± 0	360 ± 25	373 ± 30	356 ± 0	369 ± 13	367 ± 5	369 ± 13	351 ± 22	360 ± 16	355 ± 20	356 ± 0
Al_2_O_3_wt %	51.3 ± 0.6	56.2 ± 0.4	52.1 ± 0.1	54.4 ± 3.2	52.9 ± 4.3	52.1 ± 0.1	55.4 ± 1.9	59 ± 0.1	55.4 ± 1.9	51.3 ± 0.6	51.2 ± 0.8	49.9 ± 1.6	52.1 ± 0.1
CaO wt %	44.2 ± 1.1	41.1 ± 0.4	42 ± 1.2	40.9 ± 2.4	39.1 ± 1.3	42 ± 1.2	38.6 ± 1.2	37.2 ± 0	38.6 ± 1.2	44.2 ± 1.1	41.1 ± 0.3	42 ± 2.4	42 ± 1.2
SiO_2_ wt %	3.9 ± 0.4	1.7 ± 0.1	5.5 ± 1.1	4.2 ± 2.4	7.5 ± 3.2	5.5 ± 1.1	5.6 ± 3.1	3.7 ± 0.1	5.6 ± 3.1	3.9 ± 0.4	7.1 ± 0.9	7.7 ± 3.9	5.5 ± 1.1
Al_2_O_3_mass (kg)	180 ± 13	216 ± 11.2	185.4 ± 0.2	195.6 ± 15.9	197.6 ± 24.2	185.4 ± 0.2	203.9 ± 0.1	216.4 ± 2.5	203.9 ± 0.1	180 ± 13	184.1 ± 5.5	177 ± 10.2	185.4 ± 0.2
CaO mass (kg)	154.8 ± 5.7	158 ± 5.8	149.5 ± 4.3	147.3 ± 12.9	145.6 ± 11.7	149.5 ± 4.3	142.1 ± 0.5	136.4 ± 2	142.1 ± 0.5	154.8 ± 5.7	147.7 ± 5.5	149 ± 7.7	149.5 ± 4.3
SiO_2_mass (kg)	13.8 ± 2.4	6.6 ± 0.1	19.7 ± 4	15.1 ± 9	27.8 ± 11.5	19.7 ± 4	21.1 ± 12	13.5 ± 0.2	21.1 ± 12	13.8 ± 2.4	25.7 ± 4.3	27.5 ± 14.7	19.7 ± 4
Al yield	90.6 ± 0.4	89.5 ± 0.2	92.7 ± 0.4	90 ± 4.4	89.3 ± 3.4	92.7 ± 0.4	83.7 ± 2.1	84.6 ± 2.2	83.7 ± 2.1	90.6 ± 0.4	91.6 ± 0.6	90.3 ± 0.9	92.7 ± 0.4
Si recovery	92 ± 0.6	86.9 ± 5.6	83.5 ± 2.3	85.6 ± 6.2	89.3 ± 9.9	83.5 ± 2.3	83.6 ± 7	88.1 ± 11.4	83.6 ± 7	92 ± 0.6	85.5 ± 2.6	94.8 ± 3.9	83.5 ± 2.3
Ca conversion	13.5 ± 0.7	12.4 ± 0.1	13 ± 0.6	13.1 ± 1.6	12.1 ± 1.2	13 ± 0.6	14.8 ± 1.8	12.5 ± 1.6	14.8 ± 1.8	13.5 ± 0.7	12.9 ± 0.2	12.9 ± 0.8	13 ± 0.6
LAl	2.7 ± 0.1	2.6 ± 0.1	3.3 ± 0.1	2.4 ± 0.6	2.5 ± 0.6	3.3 ± 0.1	1.6 ± 0	1.7 ± 0	1.6 ± 0	2.7 ± 0.1	2.9 ± 0.1	2.7 ± 0.3	3.3 ± 0.1
LCa	1.9 ± 0.2	1.8 ± 0.1	1.7 ± 0	1.8 ± 0.2	2 ± 0.2	1.7 ± 0	1.6 ± 0	2.2 ± 0	1.6 ± 0	1.9 ± 0.2	1.7 ± 0	1.9 ± 0.1	1.7 ± 0
LSi	0.03 ± 0	0.01 ± 0	0.04 ± 0.01	0.03 ± 0.02	0.05 ± 0.02	0.04 ± 0.01	0.04 ± 0.02	0.03 ± 0	0.04 ± 0.02	0.03 ± 0	0.05 ± 0.01	0.05 ± 0.02	0.04 ± 0.01

a All values are given as the mean ± the standard deviation.

Similarly, the measure of SiO_2_ reduction rate is defined as Si recovery rate and calculated as
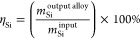
3

In addition, the conversion rate of Ca from slag phase to the metal phase is expressed as
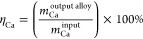
4

The element distribution between the slag phase and metal phase is evaluated as
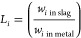
5where *w*_*i* in slag_ and *w*_*i* in metal_ represent the concentration of element *i* in slag and metal phases, respectively.

## Discussion

### Mechanism of the Aluminothermic Reduction

**Figure 9:**
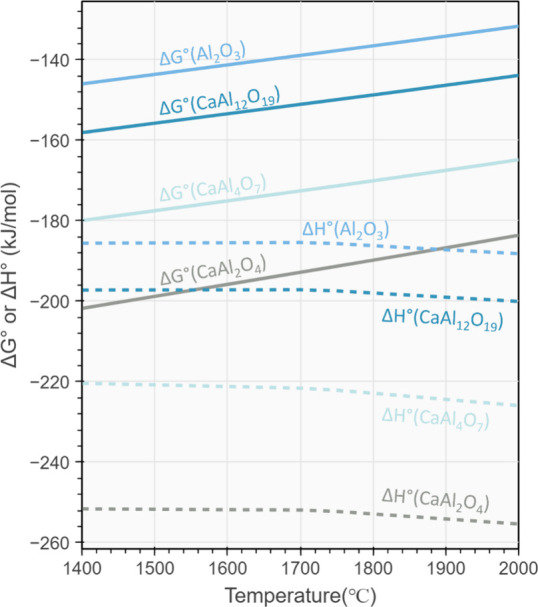
Standard Gibbs free energy change (Δ*G*°) and enthalpy change (Δ*H*°) for the possible aluminothermic reaction path with the formation of various calcium aluminate compounds. The solid lines represent the formation of Al_2_O_3_, CaAl_12_O_19_, CaAl_4_O_7_, and CaAl_2_O_4_ according to reactions 6–9. The dashed lines indicate the Δ*H*° values for the same compounds and reactions.

The reaction between Al and SiO_2_ proceeds owing to the fact that Al_2_O_3_ is thermodynamically more stable than SiO_2_, as described by reaction 6. The reaction is highly exothermic (Δ*H*°_1600 °C_ = −185.5 kJ/mol), which is crucial for the rapid progression of the reduction process. Moreover, in this work, the presence of CaO in the slag phase can significantly enhance the aluminothermic reduction process. This enhancement is primarily due to the triggering of the reactions 7–9, which are more spontaneous and exothermic compared to the baseline reaction involving only Al and SiO_2_. As illustrated in Figure 9, with the increasing presence of CaO, the standard Gibbs free energy change (Δ*G*°) for the aluminothermic reduction of SiO_2_ becomes increasingly negative, indicating enhanced spontaneity. Additionally, the enthalpy change also increases significantly, meaning the thermite reaction releases substantially more heat during the process. This causes the melt to self-heat to be maintained at high temperatures, providing two key benefits. First, the elevated temperature decreases the slag melt’s viscosity, promoting improved mass transfer conditions. Second, the heat generated reduces the overall energy consumption of the process.
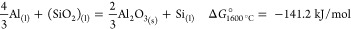
6
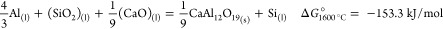
7
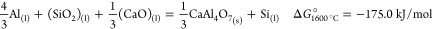
8
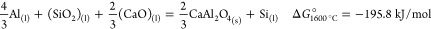
9

**Figure 10:**
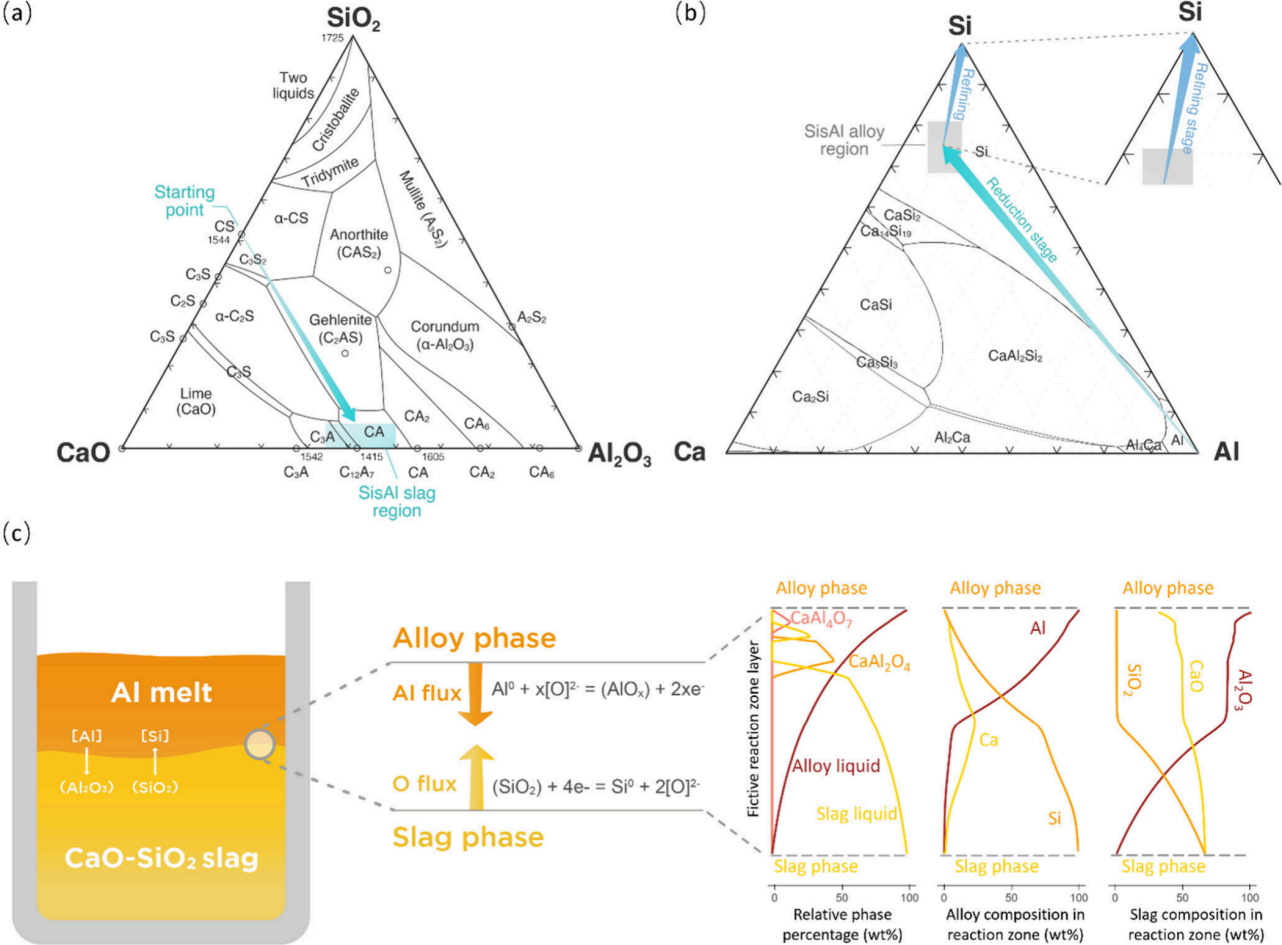
(a) Slag composition evolution path during the SisAl aluminothermic reduction process. (b) Metal phase composition evolution in the reduction stage and refining path for commercial Si production. (c) Schematic representation of the mass transfer mechanism within the reaction zone, accompanied by calculated phase percentages and compositions of the alloy and slag phases in the interfacial layer, as determined using the effective equilibrium reaction zone method at 1600 °C by Factsage.

As shown in Figure 10a, the initial composition of the slag is located at the midpoint on the CaO-SiO_2_ binary side, close to the composition of CaSiO_3_ (CS). The use of such slag as the starting point lowers the melting point of pure SiO_2_ from 1725 °C to about 1544 °C. Consequently, the molten CaO-SiO_2_ slag provides the kinetic conditions for the liquid–liquid reaction, since the high melting point and high viscosity of pure SiO_2_ may impede reaction kinetics. As the reaction progresses, the SiO_2_ content in the slag melt continuously diminishes while the Al_2_O_3_ content increases. Consequently, the slag composition shifts toward the CaO-Al_2_O_3_ portion and ultimately ends near the low-melting-point region between Ca_3_Al_2_O_6_ (C3A) and CaAl_2_O_4_ (CA) along the CaO-Al_2_O_3_ joint. Regarding the compositional evolution of the metal phase, as depicted in Figure 10b, the reduction stage produces a Si-rich alloy, typically with around 70–75 wt % Si, and 15–18 wt % Ca and 8–10 wt % Al content form accordingly. The produced SisAl alloy can be upgraded to commercial-grade Si product through a subsequent refining process.

From the perspective of mass transfer in the reaction, our previous work on the liquid–liquid reaction phenomenon observation^18^ demonstrated that the oxygen flux reaches its maximum within the first 3 min, creating a 5 μm thin reaction zone with a concentration gradient based on Al and O. As shown in Figure 10c, in the initial stage of aluminothermic reduction, due to the strong chemical affinity between the Al melt and oxygen, a counterflow of Al flux and oxygen flux are formed, leading to the rapid oxidation of Al. Simultaneously, the Si–O bonds at the slag side break quickly, causing the Si^4+^ cation to be reduced to elemental Si, which continues to supply oxygen flux to the alloy phase. Additionally, with the involvement of CaO in the slag, CaAl_*x*_O_*y*_ intermediate products may form on the side close to the alloy phase. Further insights into the reaction zone can be gained by using the effective equilibrium reaction zone (EERZ) method^26^ to simulate the mass transfer at the interfacial layer. For simplicity, the diffusion rate of all elements was assumed to be identical in the local effective reaction zone. As illustrated in Figure 10c, the calculated results show that an intermediate product layer of CaAl_4_O_7_ may appear near the alloy phase side, which is consistent with our previous experimental observations.^18^ Additionally, based on the composition variation of the alloy phase and slag phase in the reaction zone, it can be seen that the Al content in the alloy liquid decreases rapidly, while the concentration of Si is significantly higher closer to the boundary with the bulk slag melt. The thermoreduction process can also be inferred from the changes in slag composition, such as the rapid decrease in SiO_2_ corresponding to the increase in Al_2_O_3_ content, with a higher Al_2_O_3_ content observed on the alloy phase side. Moreover, it is seen that CaO is involved throughout the entire interfacial layer, reflecting the significant role of CaO in promoting the reaction.

### Reaction Analysis with Equilibrium

It is important to examine whether the pilot trials reached chemical equilibria during the operation, as it provides information regarding the efficiency of Si recovery and alumina formation. Thus, the chemical equilibria were assessed through FactSage 8.1, utilizing the databases FTLite, FToxid, and FactPS for the equilibrium calculations.

**Figure 11:**
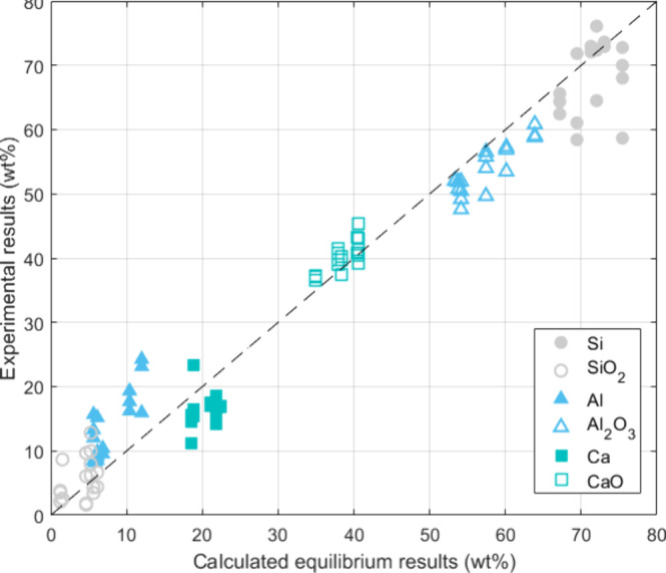
Comparison between the experimental composition and calculated equilibria.

A comparative plot of the experimental and calculated equilibrated slag and metal compositions is presented in Figure 11. It can be seen that the experimental data are in good agreement with the predicted equilibrium values. Furthermore, considering the high temperature, long reaction time, and effective stirring, the pilot trials are expected to reach or come close to their chemical equilibria. Nevertheless, slight deviations can still be observed, such as the measured Al content in the Si alloy and the CaO content in the slag, which consistently exceed the predicted values. The slight inconsistency may be attributed to database limitations in thermodynamic calculations, particularly concerning the concentrated Ca and Al content in the Si–Ca–Al ternary alloy system encountered in the SisAl process.

### Effect of Operational Parameters

As a novel process, it is crucial to understand and optimize the influence of various process parameters on the outcomes at the pilot scale. The investigation also benefits mitigation of the impact of raw material fluctuations on technical indicators. In an attempt to quantify overall impacts of different parameters, the Spearman bivariate correlation analysis,^27^ an efficient method to quantify the relationships between variables, was employed.

**Figure 12:**
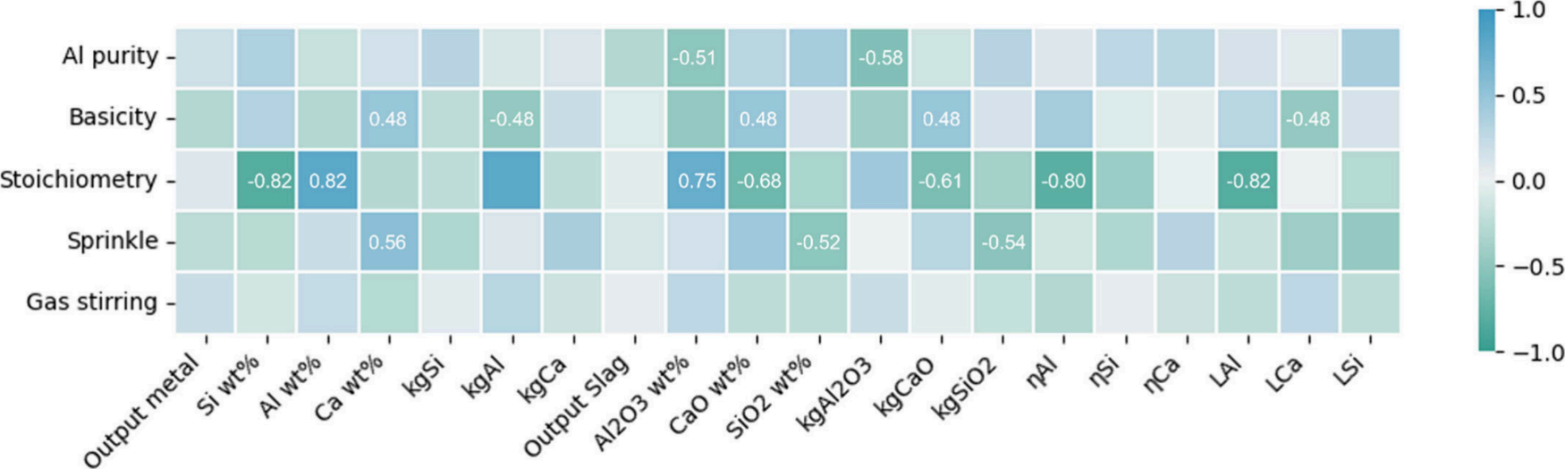
Correlation coefficient heatmap for process parameters and resultant outputs. The numbers displayed represent correlation coefficients with *p* values less than 0.05 for statistical significance.

Figure 12 presents the results of the correlation analysis, with coefficients ranging from −1 to 1, where −1 indicates a strong negative correlation and 1 indicates a strong positive correlation. The numbers displayed represent correlation coefficients with *p* values less than 0.05, signifying that there is a statistically significant correlation between the two sets of variables.

Upon examination of the results in Figure 12, it becomes evident that stoichiometry has the most statistically significant impact on the reduction results, while the influence of stirring is the least pronounced. Furthermore, for the reductant type, apart from the additional Al_2_O_3_ in dross, other operational performance evaluation indicators are not significant. Additionally, it is evident that stoichiometry and basicity significantly affect the alloy composition. Stoichiometric variation leads to completely opposite trends, with increasing Al concentrations and decreasing Si concentrations. Meanwhile, the basicity increment mainly promotes Ca content in the metal and slag. Interestingly, it also seems that different charging methods have a certain impact on the element distribution, particularly Ca. However, it is noteworthy that due to the limited size of the data set and the fact that correlation analysis only considers bivariate relationships, a certain degree of error may still exist. More detailed comparisons and analyses of the subcampaigns with controlled variables will be discussed in the following sections. The results derived from each subcampaign, listed in Table 2, provide a basis for further discussion on the impacts of these parameters.

### Effect of Reductant Type

The investigation of reductant materials was conducted through the comparison between trials T1 and T2 for the Al block, trials T7 and T8 for the Al dross, and trials T17 and T18 for the Al scrap.

**Figure 13:**
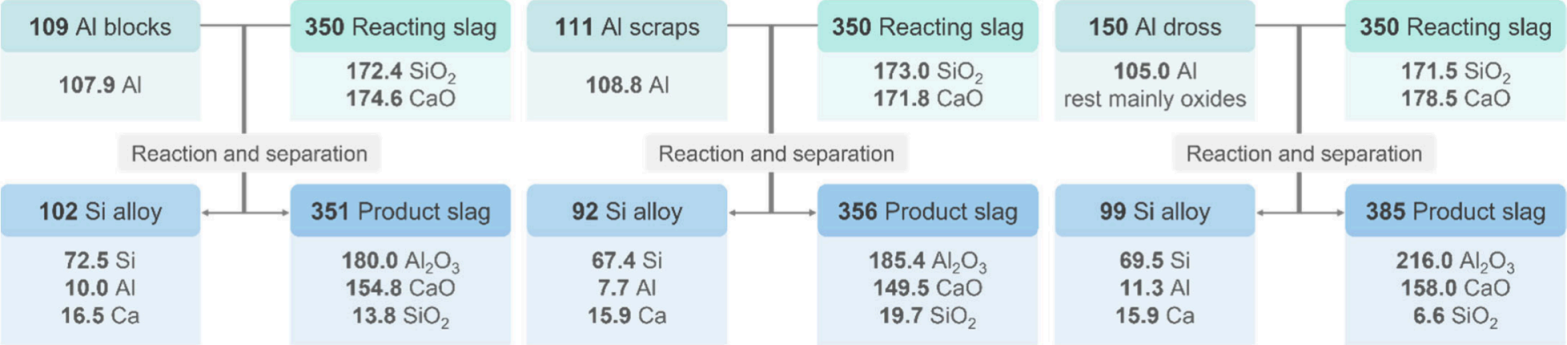
Average mass flow (kg) using neutral slag and (a) blocks, (b) scraps, and (c) dross.

The average mass flows of the key compounds are presented in Figure 13. A distinct difference is, as expected, that the dross trials led to a higher slag output, as well as a higher Al_2_O_3_ output. In the dross trials, the slag output exhibited an average Al_2_O_3_ mass of 216 ± 11.2 kg, which is significantly higher than the 180 ± 13 kg observed in the Al block trials and the 185.4 ± 0.2 kg in the scrap trials. Additionally, as shown in Figure 13, the Al_2_O_3_ content in the dross trials also exceeds that of the other trials by 4–5 wt %. The reason is attributed to the dross materials containing also a large amount of Al_2_O_3_, which directly adds up to the slag production.

**Figure 14:**
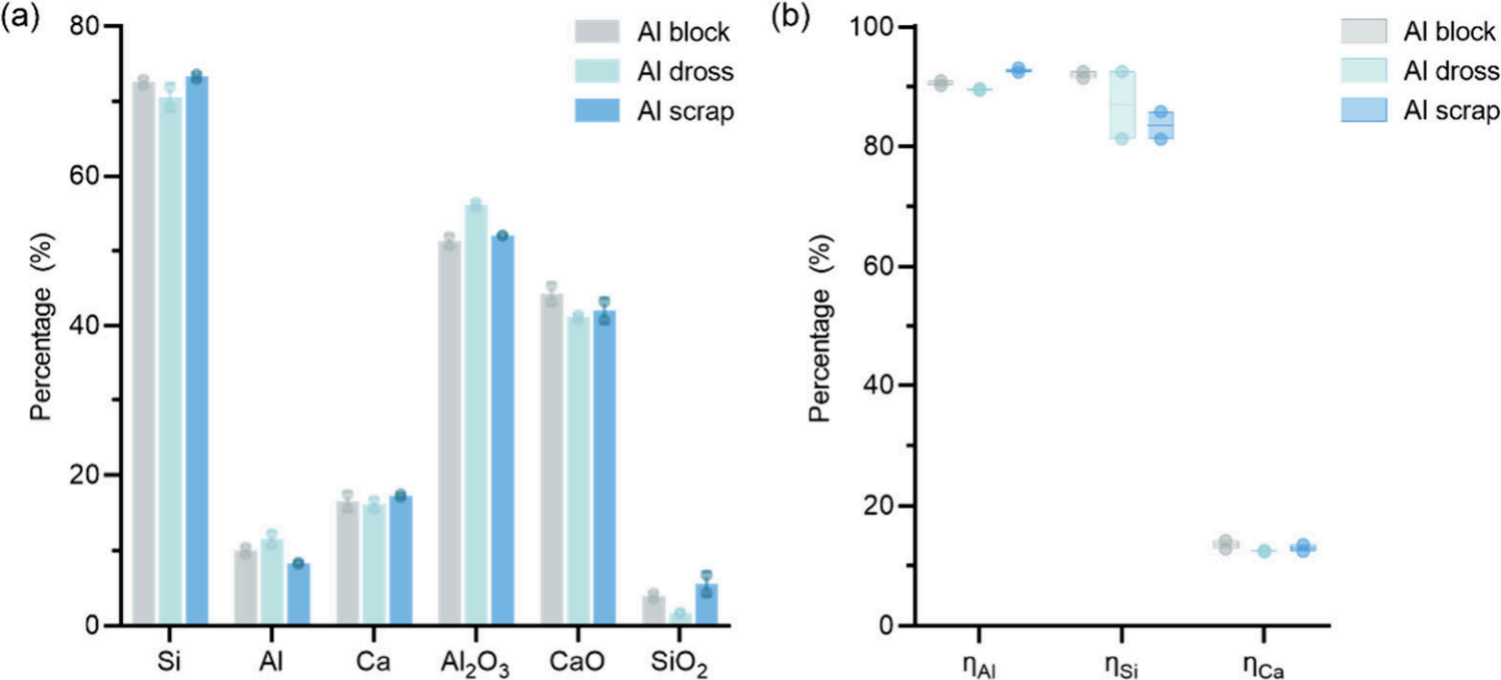
Impact assessment of reductant types (Al block, dross, and scrap) on (a) alloy and slag compositions and (b) process performance indices.

As shown in Figure 14a, apart from the Al_2_O_3_ concentration, the chemical compositions of the produced Si alloy and slags are all at the same level. For instance, the Si content ranges from 70.5 to 73.3 wt % and the Al content is within the range of 8.3 to 11.5 wt %. Furthermore, the Al yield is seen to be high at around 90% with no significant differences observed for all reductants. However, as depicted in Figure 14b, the Al block trials were slightly superior in terms of Si recovery due to a slightly higher amount of Si alloy output. One possible reason may be that the impure raw materials modify the Si distribution during the reduction. Yet, the difference is statistically insignificant, and inhomogeneity in the sampled materials may contribute to the effects. It may be concluded that the aluminothermic process demonstrates a broad flexibility in raw material input and is an efficient approach for recycling heterogeneous materials such as Al dross and scrap, producing stable products.

### Effect of Charging Methods

The influence of the two charging methods, sprinkle and co-charging, are compared across a range of processing conditions, and the benefits and potential disadvantages associated with each method are discussed below. It should be noted that the specific process durations used in the trial are related to the use of an induction furnace, which is not optimal for melting slag from an industrial point of view.

**Figure 15:**
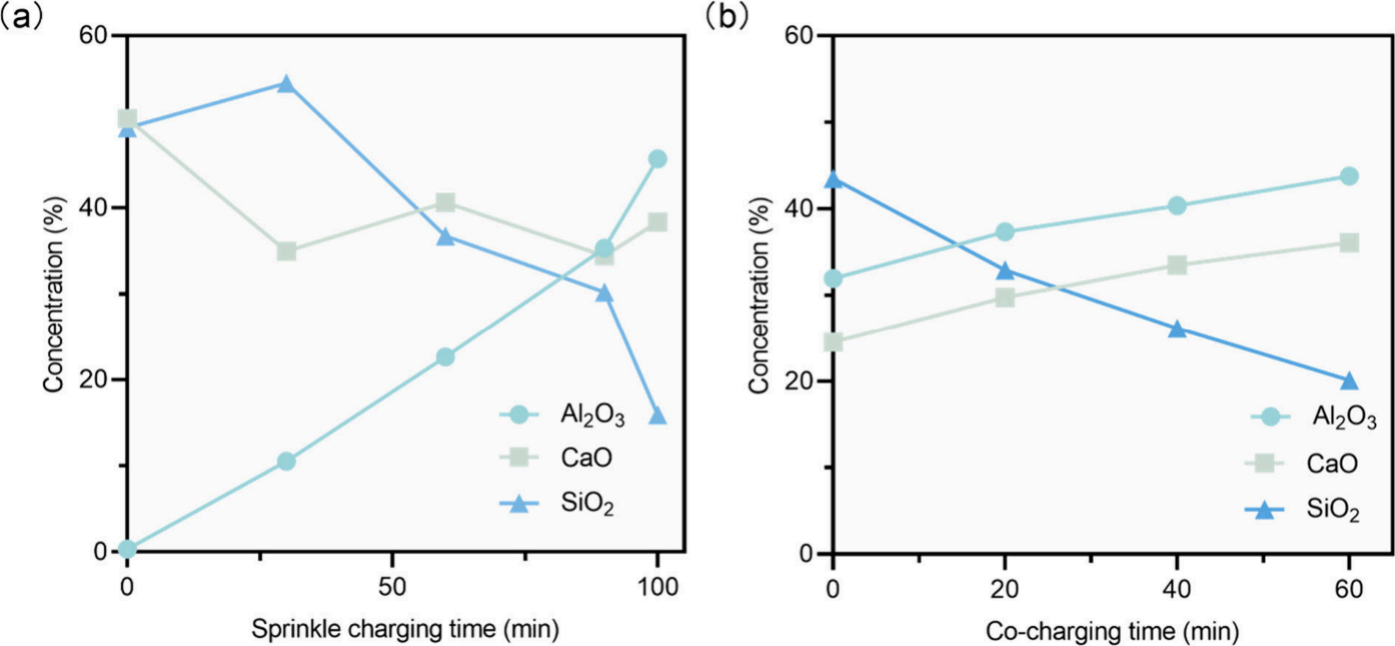
Slag composition variation with reductant charging: (a) sprinkle method and (b) co-charging method.

First, from the perspective of operation, the sprinkle method takes up to 10 h when starting from room temperature and around 6 h when the furnace is hot, i.e., directly after tapping of a previous trial, but the co-charging method led to 1–2 h shorter charging time than for the sprinkle method. Second, these two charging methods result in different compositional states for the slag and the metal melt. As shown in Figure 15, in the sprinkle method trials, the Al_2_O_3_ content in the slag increases from zero, while concurrently, the concentrations of SiO_2_ and CaO decrease. Given that the increase in slag mass due to the displacement of Al_2_O_3_ and SiO_2_ would not significantly reduce the mass fraction of CaO, it can be inferred that the main reason for the decrease in CaO concentration is the side reaction occurring between the formed Si melt and CaO. In contrast, in the co-charging method, the initial Al_2_O_3_ content in the slag is at a relatively high level, and as the charging process progresses, the concentrations of Al_2_O_3_ and CaO steadily increase, accompanied by a steady decrease in SiO_2_ concentration.

Although the temperature decreased slightly during the addition of raw materials in both methods, it remained under good control during the process due to the exothermic reactions and power adjustment. Nevertheless, in practice, the co-charging method showed signs of higher crucible degradation. This may be attributed to a more intense thermite reaction occurring during the co-charging method, or due to the consistently higher levels of Ca and Al concentration in the co-charging alloy melt, which facilitates the graphite crucible erosion due to carbide formation.^28^

**Figure 16:**
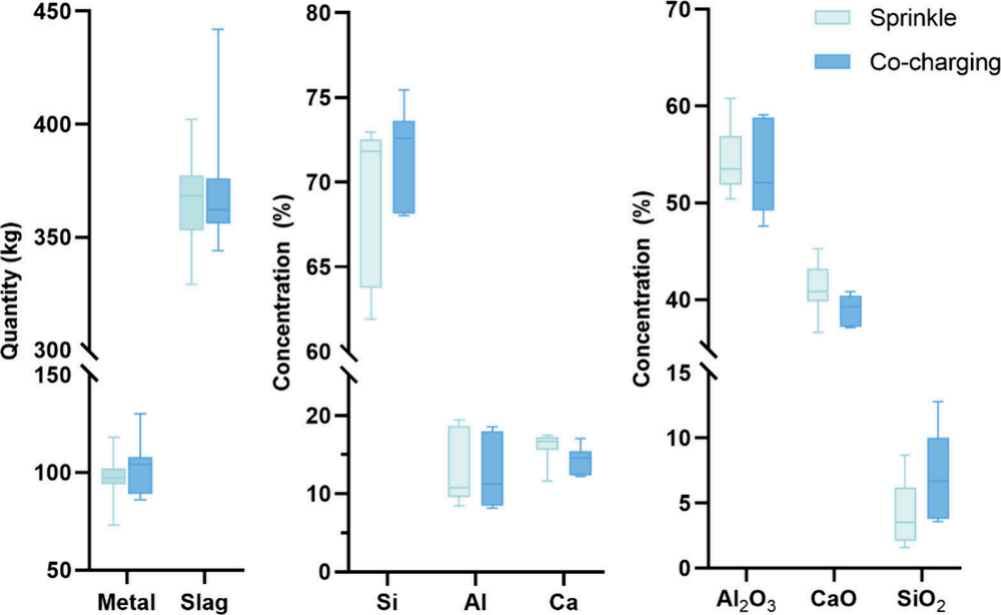
Influence of the charging methods on the quantity and composition of output products.

Based on the box chart presented in Figure 16, the two methods led to similar amount outputs of alloy and slag, but the product chemical compositions were slightly different. Compared to the co-charging method, the sprinkle trials seem to produce Si alloy with slightly lower Si content in the metal phase and associated with a higher Ca content. Consequently, it also led to a slightly lower Si recovery and higher Ca loss, as detailed in Table 2. Additionally, it appears that the SiO_2_ content in the sprinkle trials is lower than that in the co-charging trials. One important reason for this is that more overstoichiometry trials were conducted using the sprinkle method, resulting in a relatively lower Si input in these trials. However, this trend remains consistent but to a lesser extent when considering only trials 1–12 with consistent stoichiometry.

Drawing from the above discussions, it is evident that both the sprinkle and co-charging methods exhibit their own merits.

### Effect of Stoichiometry

The impact of the charging Al/SiO_2_ stoichiometry ratio on the process was evaluated by comparing the standard stoichiometry group (stoichiometry = 1, represented by trials T17 and T18) with the overstoichiometry group (stoichiometry = 1.2, represented by trials T19–T22).

**Figure 17:**
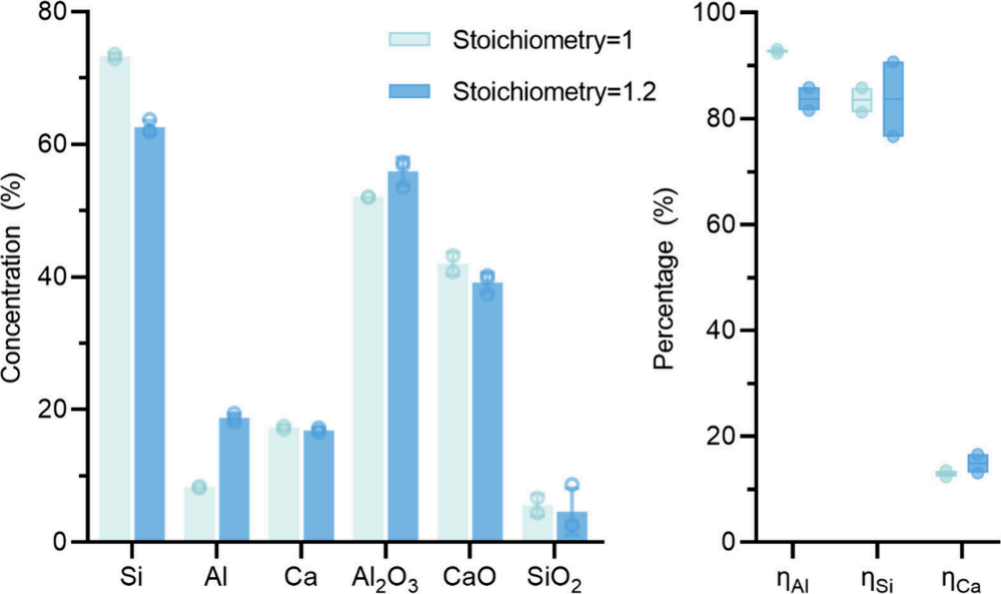
Influence of stoichiometry on the alloy and slag composition.

From the previously calculated Spearman correlation coefficients, it is evident that stoichiometry has a significant impact on the composition of the Si alloy and slag, particularly on Si, Al, Al_2_O_3_, and CaO. This trend is illustrated in Figure 17. It reveals that as stoichiometry increases, the Si content in the alloy significantly decreases from 73.3 to 62.9 wt %, while the Al content correspondingly increases from 8.8 to 18.3 wt %. In parallel, overstoichiometry trials yielded a higher Al_2_O_3_ content of 55.4 wt %, compared to the 52.9 wt % obtained in the standard stoichiometry, while the CaO content is lower than in the standard stoichiometry.

In addition to the composition variation, a significantly greater Al yield results from the overstoichiometry trials due to the presence of excess Al participating in the reaction. It suggests that the group with standard stoichiometry has a higher material utilization efficiency, as a portion of Al did not participate in the reaction in the overstoichiometry trials. On the other hand, this also indicates that the Al content in the alloy is controllable in the SisAl process, which facilitates the production of Si-Al alloys.

**Figure 18:**
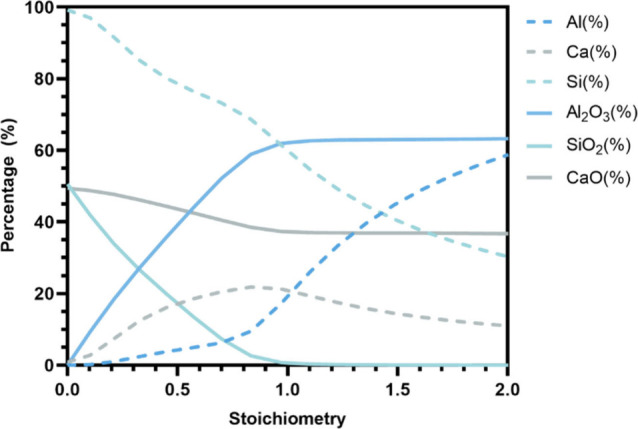
Calculated compositional evolution of the slag and metal phase under varying stoichiometries.

As Figure 18 demonstrates, the impacts of stoichiometry on the compositions of metal and slag phase can be more effectively visualized via the equilibrium calculations using Factsage 8.1. Although minor discrepancies exist, the overall trends are largely consistent. It can be seen that an increase in the stoichiometry leads to two distinct stages. Initially, with the rising amount of Al as a reductant, the SiO_2_ content in slags continuously diminishes, while the Al_2_O_3_ content sees an increment with a decline in CaO content. Simultaneously, the Si content in the metal phase continues to decrease, while the Ca and Al contents exhibit a slight increase. After the tipping point, a further increase in the stoichiometry value results in almost no further SiO_2_ reduction, but rather increases the Al content within the metal phase. However, based on the pilot study results, the appearance of the second stage is anticipated at a higher stoichiometry than the calculated values.

### Effect of Slag Basicity

To investigate the effect of slag basicity on pilot performance of the SisAl process, a comparison was drawn between trials T7 and T8 and trials T20–T22. Slags utilized in T7 and T8 were characterized by a lower basicity of 0.83, named as acidic slag, in contrast to trials T20–T22, in which a neutral slag with a basicity of 1 was used. Notably, the mass-related process indices of T21 indicated that this trial was an outlier due to the low metal output weight.

**Figure 19:**
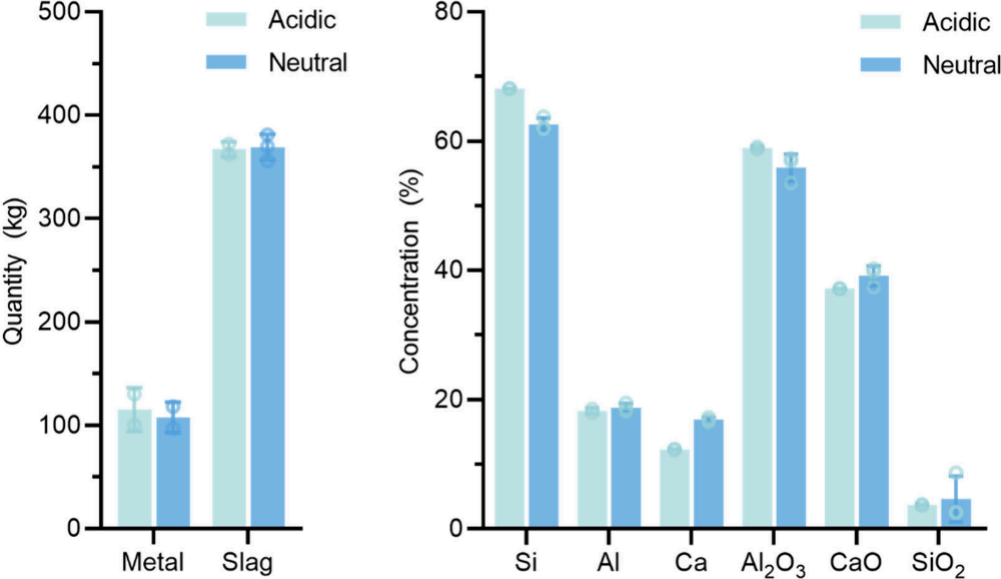
Influence of slag basicity on the alloy and slag composition.

As depicted in Figure 19, with a fixed stoichiometry, the use of acidic slag results in a larger mass of metal. Moreover, the resulting alloy from the acidic slag trials exhibits a higher Si content of 68.1 wt %, which is approximately 5 wt % higher than the 62.9 wt % in the neutral trials T20–T22. According to the Si mass content (in kg) in the output alloy, an improvement of 16% is obtained from 67.5 ± 5.7 to 78.3 ± 10.2 kg. Additionally, the acidic trials yielded a lower Ca content of 12.3 wt %, which is about 4.6 wt % lower than that of the alloys from neutral trials.

Regarding slag composition, the acidic slag also leads to a high Al_2_O_3_ content, reaching 59 wt % in the output slags, which is 4 wt % higher than that of the neutral slag trials, and 6% improvement of the total Al_2_O_3_ mass output. Moreover, the acidic trials also maintain a consistent and acceptably low level of 3.7 wt % of SiO_2_. Therefore, despite the processing indexes of the two types of slags being insignificantly different, the acidic slag with elevated Si content in the metal phase and increased Al_2_O_3_ content in the slag phase may in certain respects be a preferable option for further upscaling. However, the acidic slag may be prone to more excessive SiO fuming and higher viscosity, from an operational perspective.

### Effect of CaO-SiO_2_ Slag Prefusion

Different prefusion slag/flux charging ratios were also evaluated in the pilot scale trials. Trials T1, T2, T5, and T6 represent a high charging ratio of prefused CaO-SiO_2_ slag, while trials T17 and T18 represent a high charging ratio of flux, specifically quartz and calcined lime.

The results indicate that the prefusion trials led to a higher quantity of metal production, with 102 kg of metal produced in the high prefusion trials compared to 94 kg in the individual raw material charging trials. Given that the compositions of the produced alloys were nearly identical in these two groups, it can be inferred that the metal/slag separation was less efficient in the unfused slag trials than in those with prefused slag. As a result, the quantity of obtained Si alloy was reduced due to an increased amount of unseparated metal/slag mixtures. This uneven separation also led to deviations in the subsequent calculations, revealing an opposite trend of lower Si recovery and higher Al yield. Therefore, the data suggest that the prefusion of CaO-SiO_2_ enhances the homogenization of the slag phase and promotes more effective separation of the metal and slag.

### Effect of Stirring Methods

**Figure 20:**
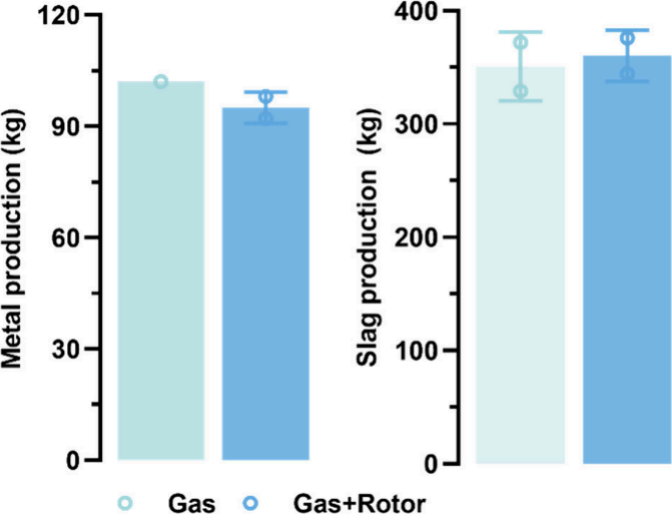
Comparison of stirring methods on product quantity.

The effects of two stirring methods, gas injection (trials T1 and T2) and rotor stirring (trials T3 and T4), were also assessed in the pilot trials (Figure 20).

In trials T3 and T4, the average metal production was only 95 kg, but trials T1 and T2 produced 102 kg. The results may indicate that the additional rotor stirring was inefficient, as it reduced the metal output. Accordingly, a lower silicon recovery rate of 85.5% was obtained compared to the 92.0% of the only gas stirring trials. In addition, it was also observed that some Al metal solidified at the top of the solidified metal cast in the rotor stirring trials, signifying incomplete and uneven mixing of the melts. In contrast, gas injection stirring exhibited commendable performance in terms of stability and efficiency, making it a more reliable choice for ensuring homogeneous mixing during the reduction process.

### Environmental Aspects

The SisAl process reveals a novel synergistic relationship between the aluminum and silicon industries: the aluminum sector supplies secondary aluminum and, in return, benefits from utilizing silicon produced via the SisAl process, exemplifying an effective industrial symbiosis. In the study by Pastor-Vallés et al.,^29^ the sustainability of the aluminothermic reduction process was benchmarked against that of the carbothermic route using LCA. One of the study’s main conclusions was that the SisAl process’s environmental impact depends strongly on the source of the aluminum input and its environmental load and allocation. Because aluminum production is linked to energy and resource consumption, using primary aluminum means that this impact may get allocated to the production of silicon. For secondary aluminum sources such as scrap, while recyclable aluminum flows will need to be diverted to silicon, system boundaries will determine its environmental load from an LCA perspective. Similarly, for dross, the environmental load is often ascribed to the Al metal that is produced. With ongoing initiatives^30,31^ to decarbonize the Al industry, it is projected that its environmental footprint will however be reduced significantly in the near future. Additionally, with optimization of process yields and resource recovery, the symbiosis between aluminum and silicon industries through the aluminothermic production of silicon may facilitate environmental gains for both industries.

## Conclusions

In the present work, a comprehensive series of pilot-scale trials was conducted for the scalability and validity of the SisAl process, an innovative approach aimed at sustainable production of Si and alumina based on side streams from the silicon and aluminum industries. Valuable insights were provided into operational efficiency for potential industrialization. The primary findings of our research can be summarized as follows.

(1) The SisAl process has proven to be a robust and stable process, as evidenced by the high similarity in the chemical composition of produced Si alloy and CaO-Al_2_O_3_ based slag. In the pilot-scale trials, key process performance indicators remaining consistent and controllable under varying process conditions.
(2) A variety of reductants, including Al blocks, dross, and scrap, were investigated in the pilot trials and all yielded comparable outcomes, with alloy composition differences only within approximately 3 wt % under the same parameter conditions, demonstrating versatility of raw material selection and an effective approach for the recycling of white dross and Al scrap for materials circularity.
(3) Acidic slag exhibited higher Si alloy yield and alumina concentration in the slag than neutral slags.
(4) The composition of the produced Si alloy and slag can be effectively controlled by modifying the reaction stoichiometry. As the charged stoichiometry increases, the Al content in the produced Si alloy also increases, while the Si content correspondingly decreases.
(5) Prefusion of CaO-SiO_2_ slag enhances slag homogenization, promoting more efficient separation between the metal and slag phases. Stirring via gas injection without rotor stirring demonstrates good stability and higher Si yield.
(6) The environmental impact of the SisAl process for silicon production may depend on the source, type, and emission allocation of aluminum reductant used, with secondary flows like scrap or dross being more sustainable than primary aluminum. Optimizing operational parameters can improve yields and reduce overall environmental and economic costs.
(7) Future research should include assessment of the process feasibility at a larger scale, along with further refinement of the products, underlying mechanism investigation, and comprehensive optimization of the process parameters.
